# Complete Genome Sequence of Elephant Endotheliotropic Herpesvirus 4, the First Example of a GC-Rich Branch Proboscivirus

**DOI:** 10.1128/mSphere.00081-15

**Published:** 2016-06-15

**Authors:** Paul D. Ling, Simon Y. Long, Angela Fuery, Rong-Sheng Peng, Sarah Y. Heaggans, Xiang Qin, Kim C. Worley, Shannon Dugan, Gary S. Hayward

**Affiliations:** a Baylor College of Medicine, Houston, Texas, USA; b Viral Oncology Program, The Johns Hopkins School of Medicine, Baltimore, Maryland, USA; c The Human Genome Sequencing Center, Houston, Texas, USA; UNC-Chapel Hill

**Keywords:** elephant endotheliotropic herpesviruses, *Elephas maximus* calf, G-plus-C nucleotide content bias, acute hemorrhagic disease, evolutionary divergence, trunk wash shedding

## Abstract

A novel group of mammalian DNA viruses called elephant endotheliotropic herpesviruses (EEHVs) belonging to the *Proboscivirus* genus has been associated with nearly 100 cases of highly lethal acute hemorrhagic disease in young Asian elephants worldwide. The complete 180-kb genomes of prototype strains from three AT-rich branch viruses, EEHV1A, EEHV1B, and EEHV5, have been published. However, less than 6 kb of DNA sequence each from EEHV3, EEHV4, and EEHV7 showed them to be a hugely diverged second major branch with GC-rich characteristics. Here, we determined the complete 206-kb genome of EEHV4(Baylor) directly from trunk wash DNA by next-generation sequencing and *de novo* assembly procedures. Among a total of 119 genes with an overall colinear organization similar to those of the AT-rich EEHVs, major features of EEHV4 include a family of 26 paralogous 7xTM and vGPCR-like genes plus 25 novel or missing genes. The genome also contains an unusual distribution of tracts of 5 to 11 successive A or T nucleotides in intergenic domains between the mostly much higher GC content protein coding regions. Furthermore, an extremely high GC-rich bias in the third wobble position of codons clearly delineates the coding regions for many but not all proteins. There are also two novel captured cellular genes, including a C-type lectin (vECTL) and an O-linked acetylglucosamine transferase (vOGT), as well as an unusually large and complex Ori-Lyt dyad symmetry domain. Finally, 30 kb from a second strain proved to include three small chimeric domains, indicating the existence of distinct EEHV4A and EEHV4B subtypes.

**IMPORTANCE** Multiple species of herpesviruses from three different lineages of the *Proboscivirus* genus (EEHV1/6, EEHV2/5, and EEHV3/4/7) infect both Asian and African elephants, but lethal hemorrhagic disease is largely confined to Asian elephant calves and is predominantly associated with EEHV1. Milder disease caused by EEHV5 or EEHV4 is being increasingly recognized as well, but little is known about the latter, which is estimated to have diverged at least 35 million years ago from the others within a distinctive GC-rich branch of the *Proboscivirus* genus. Here, we have determined the complete genomic DNA sequence of a strain of EEHV4 obtained from a trunk wash sample collected from a surviving Asian elephant calf undergoing asymptomatic shedding during convalescence after an acute hemorrhagic disease episode. This represents the first example from among the three known GC-rich branch *Proboscivirus* species to be assembled and fully annotated. Several distinctive features of EEHV4 compared to AT-rich branch genomes are described.

## Abstract (precis)

Multiple species of herpesviruses from three different lineages of the *Proboscivirus* genus (EEHV1/6, EEHV2/5, and EEHV3/4/7) infect both Asian and African elephants, but lethal hemorrhagic disease is largely confined to Asian elephant calves and is predominantly associated with EEHV1. Milder disease caused by EEHV5 or EEHV4 is being increasingly recognized as well, but little is known about the latter, which is estimated to have diverged at least 35 million years ago from the others within a distinctive GC-rich branch of the *Proboscivirus* genus. Here, we have determined the complete genomic DNA sequence of a strain of EEHV4 obtained from a trunk wash sample collected from a surviving Asian elephant calf undergoing asymptomatic shedding during convalescence after an acute hemorrhagic disease episode. This represents the first example from among the three known GC-rich branch *Proboscivirus* species to be assembled and fully annotated. Several distinctive features of EEHV4 compared to AT-rich branch genomes are described

## INTRODUCTION

The deaths of 62 young Asian elephants with acute hemorrhagic disease in North America and Europe have been attributed to rapidly developing uncontrolled primary systemic infections by members of a novel genus of mammalian herpesviruses, designated elephant endotheliotropic herpesviruses (EEHVs) (1–5). At least 43 additional lethal cases have also been confirmed by DNA tests within Asian range countries, including 16 published examples from India, Thailand, Cambodia, and Laos (6–8). Seven major genotypes, named EEHV1 to EEHV7, which all qualify as distinct species within the *Proboscivirus* genus, have been identified within North American elephants (1, 2, 9–12), although EEHV1A has been associated with the majority of lethal cases. Overall, some 46 highly divergent strains of EEHV1A have been identified by selective PCR sequencing-based gene subtyping at multiple loci. Only 10 examples of EEHV1B plus a smaller number of EEHV4 and EEHV5 viruses have also all been associated with systemic disease in Asian elephants (*Elephas maximus*), whereas three others, EEHV2, EEHV3, and EEHV6, were detected in the few rare disease cases in zoo African elephant (*Loxodonta africana*) calves (9–11). The latter three viruses as well as EEHV7 have also all been detected as quiescent infections in lung nodules from healthy adult African elephants (12). Just 12 young captive Asian elephants with confirmed EEHV1 DNA-positive systemic disease that had been treated with human antiherpesvirus drugs are considered survivors (9, 13, 14).

Until recently, it seemed that EEHV1A and its less common partially chimeric variant EEHV1B (15–18) were the predominant viruses that Asian elephants might be encountering. However, routine testing between 2011 and 2015 within the most closely monitored U.S. zoo herd (which consists of just eight individual Asian adults and calves) has now also detected episodes in which multiple herdmates underwent sequential mild primary viremic infections with subsequent trunk wash shedding by first EEHV5 (19) and then later EEHV4 (20). Because at least four different EEHV1A strains had been documented in lethal cases at this same facility within the previous 15 years and most of this herd had already been observed to undergo subclinical infections by strains of EEHV1A or EEHV1B or both either several years earlier or later (21–23), we conclude that it is likely that most Asian elephants eventually become infected with multiple EEHV species and subtypes. The relative timing and order of these primary EEHV infections are expected to have major impacts on the levels of immune protection to disease caused by the others.

Although no probosciviruses have yet been grown in cell culture, the complete genomes of four reference strains of AT-rich branch Asian elephant EEHVs have been determined previously directly from necropsy tissue, including two of EEHV1A and one each of EEHV1B and EEHV5A (24–26). Therefore, we wished to take the opportunity that this latest EEHV4 episode provided to learn more about the genetic relationships among the different EEHV lineages and species by determining the complete genomic DNA sequence of EEHV4 strain Baylor as the prototype example of a GC-rich branch proboscivirus. In the accompanying paper (27), we compare and contrast the genomes of these two major branches of the *Proboscivirus* genus as well as describe a number of additional characteristic novel features of the entire group.

## RESULTS

### Assembly of the complete 206-kb EEHV4(Baylor) genome sequence.

The intact genomes of four prototype Asian strains have all been assembled by random high-throughput and *de novo* assembly approaches directly from high-quality necropsy tissue DNA obtained from young elephants that died of acute hemorrhagic disease (24–26). For EEHV4(Baylor), we instead used a trunk wash pellet DNA sample with a high measured ratio of viral to host cell DNA. From a total of 420 million 100-bp-long runs of raw data, close to half resembled African elephant DNA and were filtered out before the remainder were assembled *de novo* by the Velvet program using a variety of different k-mer size parameters. Four independent assembled contig libraries that were searched for matches to EEHV1(Kimba) genomic DNA produced results of between one and four contigs each, with the largest being 202,155 bp. There were three small gaps in the data overall, each of which was repaired by Sanger PCR sequencing and proved to be located in internal repetitive regions either within E34 (ORF-C) or in the predicted Ori-Lyt locus between U41 (major DNA-binding protein [MDBP]) and U42 (MTA) or close to the right terminus. Because of this being intracellular and not virion-derived DNA, the final results in each case were identical contiguous circular genomes of 205,896 bp (average coverage of 110-fold).

To generate a linear version, we arbitrarily defined a G10 tract at the beginning of the original largest contig as the left end of the EEHV4(Baylor) genome. Importantly, two regions mapping very close to the right end contained distinct sets of potential packaging signal motifs, of which the first matched at 45 out of 54 nucleotides (83%) to both copies of the terminal repeats of EEHV1A(Emelia) and EEHV1A(Raman) and the second matched at 103 of 144 positions (72%) to all three copies of the terminal repeat “a” sequence of herpes simplex virus 1 KOS [HSV-1(KOS)]. The extreme left side of the genome (outside gene E1) contains several segments, including a set of 17-bp tandem repeats as well as a cluster of 8-bp palindromic cyclic AMP (cAMP) response elements (CREs) that have high-level homology to repetitive sequences present within the terminal repeats of the other EEHV genomes. Therefore, there is clear evidence that both ends of our assembled EEHV4 genome map within the predicted terminal repeat and lie close to the legitimate physical ends of the EEHV1A, EEHV1B, and EEHV5 genomes as determined by Wilkie et al. (25, 26). Furthermore, on the far right side of the EEHV4(Baylor) genome there is a 5.3-kb noncoding segment lying outside and immediately upstream of the gene encoding the U44 (ORF-L) transcription factor-like protein. This region is similar in size to the nearly 7 kb of noncoding DNA (which includes all of one copy of the 2.9-kb terminal repeat) at that position in the linear AT-rich branch genomes of the work of Wilkie et al. (25, 26). Based on the fact that we could assemble a single intact contig with terminal repeat sequences near both expected ends, we deduced that this must represent the entire EEHV4(Baylor) genome.

### Global features and initial comparisons with other EEHV genomes.

**FIG 1:**
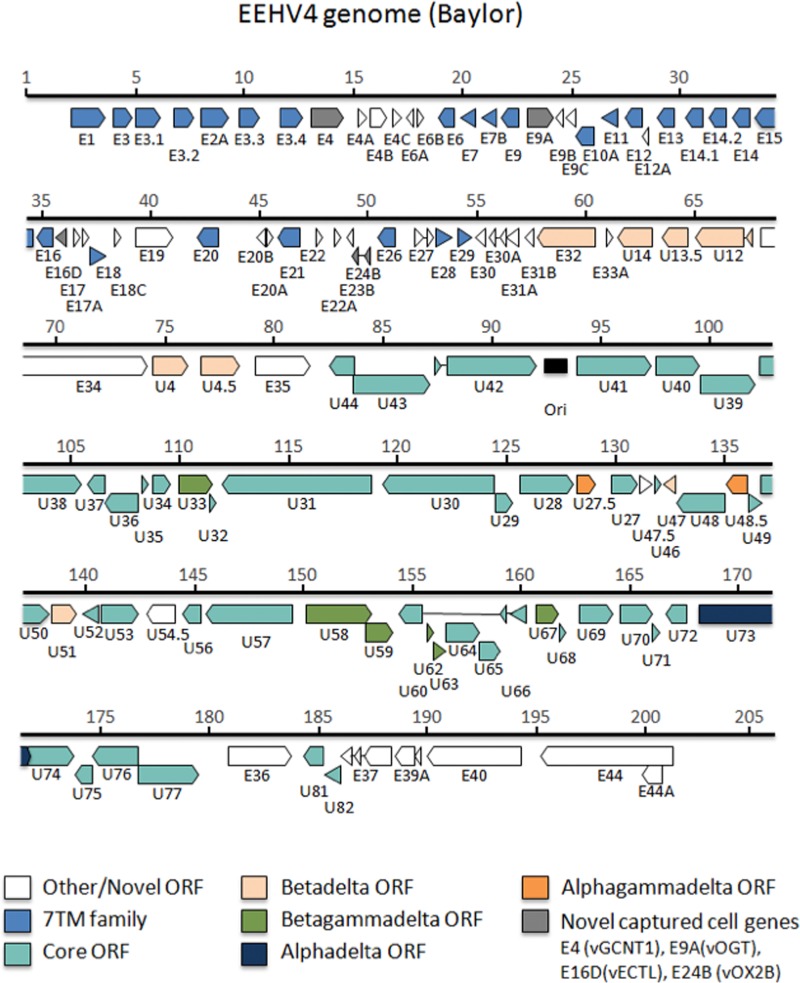
Annotated physical gene map of the complete EEHV4(Baylor) genome. The intact 206-kb EEHV4B(Baylor) genome determined here (GenBank accession no. KT832477) is depicted to scale. The relative sizes and orientations of all predicted open reading frames (ORFs) are indicated by horizontal arrows. Gene nomenclature is shown below each of the ORFs. The color key indicates groups of ORFs shared between all herpesviruses or subsets of herpesvirus subfamilies or multiple paralogues of repetitive gene families. Gray arrows indicate captured cellular genes, and white arrows denote novel genes that do not have obvious orthologues outside of the probosciviruses. Thin lines connecting arrows indicate introns. The position of the putative lytic replication origin is marked by a black rectangle.

The genome of EEHV4(Baylor) contains 119 open reading frames (ORFs) arranged as shown in the map in Fig. 1. A full listing of the names, sizes, and map coordinate positions for all designated protein coding ORFs arranged from left to right across the EEHV4(Baylor) genome oriented in the same direction as in our prototype EEHV1(Kimba) is presented in Table 1. These data include comparative information relative to the intact genomes of EEHV1A(Kimba) and EEHV1A(Raman), EEHV1B(Emelia), and EEHV5A(Vijay) to indicate all of the genes present in EEHV4 that are not found in the others, as well as all genes present within the others that are missing from EEHV4. Table 1 also shows the GC content of each ORF in EEHV4 and whether the gene is assigned to a gene family and its status as a common core herpesvirus gene or is shared between subsets of the alphaherpesvirus, betaherpesvirus, and gammaherpesvirus subfamilies or is unique to the *Proboscivirus* genus. Because we are proposing here that the probosciviruses have numerous novel biological properties and genetic and evolutionary features that may justify their future designation as a new deltaherpesvirus subfamily of mammalian herpesviruses, to reduce possible confusion later on we have adopted an interim dual nomenclature of either *p* or δ when referring to unique features of the *Proboscivirus* gene and protein sets in a phylogenetic context.

**TABLE 1:** Gene content and major features of the complete 205,896-bp EEHV4(Baylor) genome*^h^,^i^*

Gene name and orientation	Protein name	Type	Family or status	Position coordinates	% GC content	Protein size (aa)	% amino acid identity (% length matched) to:				Note or comment
							EEHV1A Kimba	EEHV1A Raman	EEHV1B Emelia	EEHV5 Vijay	
TR	3.5× 22-bp			340–420			Related to multimerized 17-bp repeats in TR of EEHV1A/1B/5				
TR	Regulatory motifs			1070–1410			All have a cluster of 6–9× palindromic (8-bp) CREB-binding sites				
Nil	vFUT9		Novel				E47	EE63	EE63	EE63	Absent in EEHV4^a^
Nil		7xTM	Novel				Nil	Nil	Nil	EE62B	Unique to EEHV5
Nil			IgFam				Nil	Nil	Nil	EE62A	Unique to EEHV5
Nil	vGPCR7	7xTM	E3fam				E48	EE62	Nil	EE62	Absent in EEHV4
Nil			E49fam				E49	EE61	Nil	Frag	Absent in EEHV4
Nil	vIgF1		Novel				E50	EE60	Nil	Nil	Absent in EEHV4, -5 outside the probosciviruses
Nil	vGPCR8	7xTM	E3fam				Frag	Frag	EE59	Nil	Absent in EEHV4
Nil			E49fam				E51	EE58	EE58	Nil	Absent in EEHV4
Nil	vIgF2		IgFam				E52	EE57	Frag	Nil	Absent in EEHB4
Nil			E49fam				Nil	Nil	EE56	Nil	Absent in EEHV4
Nil			IgFam				Nil	Nil	EE55	Nil	Absent in EEHV4
Nil			IgFam				Nil	Nil	EE54	Nil	Absent in EEHV4
Nil	vIgF2.4		IgFam				Frag	EE53	EE53	Nil	Absent in EEHV4
Nil	vIgF2.5		IgFam				E53	EE52	EE52	EE52	Absent in EEHV4
Nil	vOX2-1		Novel				E54	EE51	EE51	EE51	Absent in EEHV4
Nil	vIgF3		IgFam				E55	EE50	EE50	EE50	Absent in EEHV4
Nil	vCD48		IgFam				Nil	Nil	Nil	EE49D	Unique to EEHV5
Nil			IgFam				Nil	Nil	Nil	EE49C	Unique to EEHV5
Nil	vCD48		IgFam				Nil	Nil	Nil	EE49B	Unique to EEHV5
Nil			IgFam				Nil	Nil	Nil	EE49A	Unique to EEHV5
E1, F		7xTM	E3fam	2061–3608	69	515	25 (45)	EE49	EE49	EE49	N-term S/T extended
Nil	Cys-rich	7xTM	E3fam				E2	EE48	EE48	EE48	Absent in EEHV4
E3, F	vGPCR6	7xTM	E3fam	3981–4928	68	315	30 (70)	EE47	EE47	EE47	Match to RAIP3 or C-5-Afam
E3.1, F	vGPCR6.1	7xTM	E3fam	4991–6169	62	392	28 (60)	EE45	EE45	EE45	Unique to EEHV4
E3.2, F	vGPCR6.2	7xTM	E3fam	6747–7727	67	328	30 (45)	EE45	EE45	EE45	Unique to EEHV4
E2A, F		7xTM	Novel	8015–9295	58	426	Nil	Nil	Nil	Nil	Unique to EEHV4; S/T dom
E3.3, F	vGPCR6.3	7xTM	E3fam	9714–10721	50	335	34 (41)	EE45	EE45	EE45	Unique to EEHV4
E3.4, F	vGPCR6.4	7xTM	E3fam	11637–12674	56	345	38 (69)	EE45	EE45	EE45	Unique to EEHV4
E4, F	vGCNT1	AcTransf	Novel	13026–14642	63	538	61 (68)	EE46	EE46	EE46	—^b^
Nil	vGPCR5	7xTM	E3fam				E5	EE45	EE45	EE45	Absent in EEHV4
Nil			Novel				E5A	EE44	EE44	Nil	Short, memb, absent EEHV4
Nil	vCD48		IgFam				Nil	Nil	Nil	EE44A	Unique to EEHV5
**E4A, F**			**Novel**	**15171–15653**	**44**	**160**	**Nil**	**Nil**	**Nil**	**Nil**	**Unique to EEHV4**
E4B, F			Novel	15712–16578	56	288	Nil	Nil	Nil	Nil	Unique to EEHV4
E4C, F			Novel	16779–17234	54	151	Nil	Nil	Nil	Nil	Unique to EEHV4
E6A, C	E27ex1		Novel	17811–17398	57	137	E27	EE20	Nil	EE20	35% (44%) match to E27
E6B, F			Novel	17978–18256	49	92	Nil	Nil	Nil	Nil	Unique to EEHV4
E6, C		7xTM	E6fam	19679–18855	60	274	33 (89)	EE43	EE43	EE43	
Nil, C	vCXCL2?		E7A					Nil	Nil	Nil	Absent in EEHV1B/4/5
E7, C		7xTM	E6fam	20663–19956	55	235	32 (84)	EE42	EE42	EE42	
**E7B, C**		**7xTM**	**Novel**	**21647–20871**	**26**	**258**	**Nil**	**Nil**	**Nil**	**Nil**	**Unique to EEHV4**
**Nil**		**7xTM**	**E6fam**				**E8**	**EE41**	**EE41**	**EE41**	**Absent in EEHV4**
**E9, C**		**7xTM**	**E6fam**	**22653–21757**	**27**	**298**	**34 (18)**	**EE40**	**Frag**	**EE40**	**Match to C-term EE40 only (esp EEHV5)**
**E9A, F**	**vOGT**	**AcTransf**	**Novel**	**22993–24237**	**42**	**414**	**Nil**	**Nil**	**Nil**	**Nil**	**Unique to EEHV4**^c^
**E9B, C**	**(Truncated)**		**Novel**	**24664–24281**	**23**	**127**	**Nil**	**Nil**	**Nil**	**EE39/40**	**Match to EEHV5 N-term16-aa EE39/40 only**
**E9C, C**			**Novel**	**25233–24718**	**27**	**171**	**Nil**	**Nil**	**Nil**	**Nil**	**Unique to EEHV4**
E10A, C		7xTM	E6fam	26078–25197	61		Nil	Nil	Nil	25 (46)	Matches central EE40(EEHV5) only
Nil		7xTM	E6fam				E10	EE39	EE39	EE39	Absent in EEHV4
E11, C		7xTM	E6fam	27127–26363	61	254	36 (96)	EE38	EE38	EE38	
E12, C		7xTM	E6fam	28314–27457	60	285	34 (77)	EE37	EE37	EE37	
E12A, C			Novel	28547–28281	60	88	Nil	Nil	Nil	Nil	Unique to EEHV4
E13, C		7xTM	E6fam	29780–28965	58	271	52 (88)	EE36	EE36	EE36	
E14.1, C		7xTM	E14fam	31024–30215	59	269	27 (91)	Nil	Nil	Nil	Duplication of E14
E14.2, C		7xTM	E14fam	32191–31280	52	303	24 (77)	Nil	Nil	Nil	Duplication of E14
E14, C		7xTM	E14fam	33228–32419	57	269	26 (79)	EE35	EE35	EE35	
E15, C	vGPCR4	7xTM	E15fam	34435–33425	58	336	34 (86)	EE34	EE34	EE34	26% (49%) match to Lox C-5-C
E16, C		7xTM	E14fam	35571–34768	57	267	40 (97)	EE33	EE33	EE33	
Nil			Novel				E16C	Nil	Nil	Nil	Conserved in EEHV1 and EEHV5
Nil			Novel				E16A/B	Nil	Nil	Nil	Spliced; unique to EEHV1A/B
**E16D, C**	**vECTL**		**Novel**	**36141–35584**	**45**	**185**	**Nil**	**Nil**	**Nil**	**Nil**	
**E17, F**	**E27ex1**		**Novel**	**36491–36826**	**45**	**111**	**37 (99)**	**EE32**	**EE32**	**EE32**	**Match to E27, 30% (50%)**
E17A, F			Novel	36944–37252	63	102	Nil	Nil	Nil	Nil	Unique to EEHV4
Nil			Novel				Nil	Nil	Nil	EE32A	Unique to EEHV5
E18, F		7xTM	E18fam	37215–38018	57	267	32 (70)	EE31	EE31	EE31	Related to E28 by 30% (51%)
Nil			Novel				E18B	EE30A	EE30A	EE30A	Absent in EEHV4
Nil			Novel				E18A	EE30	EE30	EE30	Absent in EEHV4
E18C, F			Novel	38433–38720	59	94	Nil	Nil	Nil	Nil	Unique to EEHV4
E19, F	ORF-F2		U54.5fam	39303–41042	68	579	52 (89)	EE29	EE29	EE29	25% (77%) to ORF-F1
E20, C	vGPCR4A	7xTM	E15fam	43188–42154	57	344	46 (84)	EE28	EE28	EE28	23% (67%) match to Lox RAIP3
E20B, C			Novel	45323–44904	65	139	Nil	Nil	Nil	Nil	Unique to EEHV4
E20A, F			Novel	45338–45664	61	108	43 (33)	EE27	EE27	EE27	
E21, C	vGPCR4B	7xTM	E15fam	46925–45810	59	371	35 (76)	EE26	EE26	EE26	27% (35%) match to Lox RAIP3
E22, F			Novel	47646–47921	49	91	48 (96)	EE25	EE25	EE25	
E22A, F			Novel	48621–48730	53	79	46 (48)	EE24	EE24	EE24	
E23B, C			Novel	48993–49325	57	110	Nil	Nil	Nil	Nil	Unique to EEHV4
E24B, C	vOX2-Bex2		Novel	49596–49250	56	132	E54	EE51	EE51	EE51	
	vOX2-Bex1		Novel	50001–49950							Short first exon
Nil	vOX2-3						E24	EE23	EE23	EE23	Absent in EEHV4
Nil	vOX2-V (E23A)						Nil	Nil	Nil	EE22A	Unique to EEHV5
Nil	vOX2-2						E25	EE22	EE22	EE22	Absent in EEHV4
E26, C	vGPCR3	7xTM	E3fam	51287–50418	50	289	42 (92)	EE21	EE21	EE21	Match to ChemR C-5-C
E27, F	E27ex1	E27	Novel	52138–52606	55	245	57 (58)	EE20ex1	EE20ex1	EE20ex1	Related to E6A, E17
	E27ex2		Novel	52785–53053	59		Nil	Nil	Nil	Nil	Unrelated to EE20ex2
E28, F		7xTM	E18fam	53115–53861	54	248	44 (90)	EE19	EE19	EE19	Related to E18 by 30% (51%)
E29, F		7xTM	Novel	54092–54781	55	229	42 (91)	EE18	EE18	EE18	
E30, C			Novel	55453–54911	55	180	<15	EE17	EE17	EE17	Acidic similarity only
**E30A, C**	**E30Aex2**		**Novel**	**55883–55534**	**42**	**133**	**Nil**	**Nil**	**Nil**	**Nil**	**Unique to EEHV4**
	**E30Aex1**		**Novel**	**56146–56095**	**40**		**Nil**	**Nil**	**Nil**	**Nil**	**Unique to EEHV4**
Nil							E31	EE16	EE16	EE16	Absent in EEHV4
E31A, C			Novel	56923–56321	56	200	35 (46)	EE15	EE15	EE15	Only N-term cons
E31B, C			Novel	57113–56610			Nil	Nil	Nil	Nil	Unique to EEHV4
E31C, C	E31Cex		Novel	57646–57182	61	136	65 (12)	EE14	EE14	EE14	No ATG, splice to E32?
E32, C	U14.5		βδ?	60473–57717	60	918	45 (82)	EE13	EE13	EE13	
Nil, F							E33	EE12A	Frag	Nil	Unique to EEHV1A
E33A			Novel	60991–61236	50	81	37 (59)	EE12	EE12	EE12	
U14, C	U14		βδ	63078–61408	59	556	37 (75)	U14	U14	U14	
U13.5, C	U13.5		βδ	64742–63444	52	432	76 (54)	UL34	UL34	UL34	
U12, C	vGPCR2ex2	7xTM	βδ	67327–65060	57	783	50 (53)	U12	U12	U12	
	vGPCR2ex1			67537–67454			56 (100)	U12	U12	U12	Short first exon
E34, F	ORF-C		Novel	68025–74276	59	2,083	42 (16)	U11	U11	U11	Only N-term cons in EEHV1, and -5
U4, F	U4	U4	βδ	74389–76053	61	554	58 (94)	U4	U4	U4	24% (38%) HHV6 U4
U4.5, F	ORF-B	U4	βδ	76687–78444	63	585	59 (93)	EE11	EE11	EE11	24% (30%) U4
E35, F	ORF-A		Novel	79137–81659	63	840	51 (46)	EE10	EE10	EE10	
U44, C	U44		Core	82518–83729	55	403	77 (23)	U44	U44	U44	Only C-term cons in EEHV1 and -5
U43, F	PRI		Core	83629–87234	59	1,201	51 (84)	U43	U43	U43	Primase subunit
U42, F	MTAex1			87495–87627			65 (51)	U42	U42	U42	Short first exon
	MTAex2		Core	87948–92020	64	1,401	52 (24)	U42	U42	U42	Posttranscriptional regulator
Ori-Lyt				92346–93325							—^d^
U41, F	MDBP		Core	93861–97376	61	1,171	63 (99)	U41	U41	U41	SS DNA binding protein
U40, F	TER2		Core	97498–99591	59	697	73 (98)	U40	U40	U40	
U39, F	gB		Core	99533–102121	57	862	64 (94)	U39	U39	U39	Env glycoprotein B
U38, F	POL		Core	102273–105527	62	1,084	65 (99)	U38	U38	U38	DNA polymerase
U37, C	DOC		Core	106545–105740	59	268	64 (97)	U37	U37	U37	Docking protein
U36, C			Core	108154–106538	63	538	69 (89)	U36	U36	U36	
U35, F			Core	108266–108553	48	95	69 (98)	U35	U35	U35	
U34, F			Core	108717–109619	53	300	63 (99)	U34	U34	U34	
U33, F	CRP		βγδ	109914–111518	64	534	49 (91)	U33	U33	U33	Cys-rich protein
U32, F	SCP		Core	111409–111672	58	87	46 (67)	U32	U32	U32	Small capsid protein
U31, C	TEG-L		Core	118856–111873	65	2,321	44 (89)	U31	U31	U31	Large tegument
U30, C	TEG-S		Core	124420–119289	61	1,713	45 (53)	U30	U30	U30	Small tegument
U29, F	TRI1		Core	124423–125313	60	296	60 (98)	U29	U29	U29	Capsid triplex 1
U28, F	RRA		Core	125563–128073	61	836	68 (66)	U28	U28	U28	Ribonucleotide reductase A
U27.5, F	RRB (ORF-H)		αγδ	128212–129117	53	301	75 (99)	EE9	EE9	EE9	Ribonucleotide reductase B
U27, F	PPF		Core	129702–131023	64	437	52 (65)	U27	U27	U27	Pol processivity factor
U45.7, F	ORF-J		Novel	131035–131784	58	216	44 (33)	EE8	EE8	EE8	
**U46, F**	**gN**		**Core**	**131800–132105**	**49**	**101**	**63 (53)**	**U46**	**U46**	**U46**	**Env glycoprotein N**
**U47, C**	**gO (ORF-D)**		**Βδ**	**132837–132187**	**52**	**216**	**34 (94)**	**U47**	**U47**	**U47**	**Env glycoprotein O**
**U48, C**	**gH**		**Core**	**135086–132816**	**49**	**756**	**47 (96)**	**U48**	**U48**	**U48**	**Env glycoprotein H**
U48.5, C	TK (ORF-E)		αγδ	136101–135052	56	349	50 (87)	EE7	EE7	EE7	Thymidine kinase
U49, F			Core	136100–136801	57	233	48 (85)	U49	U49	U49	
U50, F	PAC2		Core	136620–138347	57	575	64 (99)	U50	U50	U50	Packaging
U51, F	vGPCR1	7xTM	βδ	138430–139647	56	405	42 (95)	U51	U51	U51	—^e^
U52, C			Core	140605–139832	53	257	65 (98)	U52	U52	U52	
U53, F	SCA/PRO		Core	140698–142485	60	595	49 (91)	U53	U53	U53	Scaffold protease
U54.5, C	ORF-F1		U54.5fam	144154–142715	61	479	38 (99)	U54	U54	U54	27% (95%) match to ORF-F2
U56, C	TRI2		Core	145344–144445	59	299	68 (99)	U56	U56	U56	Capsid triplex 2
U5, C	MCP		Core	149563–145511	63	1,350	71 (99)	U57	U57	U57	Major capsid protein
U58, F	vTBP		βγδ	150117–153134	61	1,005	63 (87)	U58	U58	U58	TATA-binding protein
U59, F			βγδ	152794–154152	62	452	48 (79)	U59	U59	U59	
U60, C	TERex3		Core	155504–154377	57	660	92 (99)	U60	U60	U60	Terminase subunit 1
U62, F			βγδ	155733–156005	54	90	57 (97)	U62	U62	U62	
U63, F			βγδ	155944–156546	51	200	67 (72)	U63	U63	U63	
U64, F	PAC1		Core	156527–158552	64	541	48 (63)	U64	U64	U64	Packaging
U65, F			Core	158055–159071	59	338	48 (98)	U65	U65	U65	
U66, C	TERex2		Novel	159272–159153			90 (100)	U66	U66	U66	Terminase subunit 1
	TERex1		Core	160224–159490	53		89 (99)	U66	U66	U66	Terminase subunit 1
U67, F			βγδ	160612–161742	58	376	61 (98)	U67	U67	U67	
U68, F			Core	161739–162104	51	121	66 (98)	U68	U68	U68	
U69, F	CPK		Core	162613–164232	59	539	57 (96)	U69	U69	U69	Conserved protein kinase
U70, F	EXO		Core	164529–166094	61	521	53 (97)	U70	U70	U70	Exonuclease
U71, F	MyrTeg		Core	166031–166342	56	103	41 (67)	U71	U71	U71	Myristylated tegument
U72, C	gM		Core	167655–166537	55	372	66 (93)	U72	U72	U72	Envelope glycoprotein M
U73, F	OBP (ORF-G)		αδ	168094–171645	61	1,183	65 (68)	U73	U73	U73	Origin-binding protein
U74, F	PAF		Core	171659–173830	63	723	61 (91)	U74	U74	U74	Pol-associated factor
U75, C			Core	174625–173813	63	270	54 (84)	U75	U75	U75	
U76, C	POR		Core	176750–174579	63	723	74 (78)	U76	U76	U76	Portal protein
U77, F	HEL		Core	176701–179574	63	957	79 (79)	U77	U77	U77	Helicase subunit
E36, F	ORF-M		Novel	180833–183805	65	990	62 (19)	U79	U79	U79	Env glycoprotein M (only N-term cons)
Nil	ORF-N, vCXCL1		Novel				E36A	EE6	Nil	EE6	Chemokine-like, absent in 1B, 4
U81, C	UDG		Core	185284–184277	63	335	68 (68)	U81	U81	U81	Uracil DNA glycosylase
U82, C	gL		Core	186080–185253	48	275	37 (94)	U82	U82	U82	Env glycoprotein L
**E37, C**	**ORF-Oex3**		**Novel**	**186570–186007**	**41**	**709**	**37 (95)**	**EE5**	**EE5**	**EE5**	
	**ORF-Oex2**		**Novel**	**186897–186677**	**40**		**<15**	**EE5**	**EE5**	**EE5**	
	**ORF-Oex1**		**Novel**	**188411–187067**	**41**		**<15**	**EE5**	**EE5**	**EE5**	
**Nil**	**ORF-Pex2**		**Novel**				**E38**	**EE4**	**EE4**	**EE4**	**Absent in EEHV4**
	**ORF-Pex1**		**Novel**				**E38**	**EE4**	**EE4**	**EE4**	**Absent in EEHV4**
**Nil**	**ORF-Qex2**		**Novel**				**E39**	**EE3**	**EE3**	**Nil**	**Absent in EEHV2, -4, -5**
	**ORF-Qex1**		**Novel**				**E39**	**EE3**	**EE3**	**Nil**	**Absent in EEHV2, -4, -5**
**E39A, C**	**ORF-Rex2**		**Novel**	**189482–188506**	**40**	**367**	**Nil**	**Nil**	**Nil**	**Nil**	**Unique to EEHV4**
	**ORF-Rex1**		**Novel**	**189690–189564**	**32**		**Nil**	**Nil**	**Nil**	**Nil**	**Unique to EEHV4**
E40, C	ORF-K		Novel	194372–189975	66	1,465	72 (16)	EE2	EE2	EE2	Only C-term cons
E44A, C	ORF-S		Novel	200795–199809	64	328	36 (84)	EE1A	EE1A	EE1A	Overlaps ORF-L
E44, C	ORF-L IE-like		Novel	201282–195226	65	2,018	52 (11)	EE1	EE1	EE1	Transcriptional regulator
TR	Palindrome			202971–203014							45-bp hairpin
TR	Packaging motifs			205612–205665							—^f^
TR	Packaging motifs			205784–205896							—^g^

a Fucosyl transferase 9 = EC 2.4.1.152.

b Acetylglucosamine transferase 1 = EC 2.4.1.1.

c UDP-β-Gal *N*-acetylglucosamine transferase 3, also known as O-linked *N*-acetylglucosamine transferase = EC 2.4.1.255.

d Complex dyad symmetry. Resemblance to alphaherpesvirus Ori-L and Ori-S as well as HHV6 Ori-Lyt, but not to cytomegalovirus Ori-Lyt, much larger than EEHV1 and EEHV5 versions, 3× 90-bp and other dyad symmetry elements with 5× OBP-binding site motifs plus 35× 20-bp AT-rich tandem repeats.

e No matches to other betaherpesvirus vGPCRs.

f 83% DNA match over 54 bp to terminal repeat motifs at 2852 to 2905 and 180311 to 180358 in EEHV1B(Emelia).

g 72% DNA match over 112 bp to terminal repeat motifs present in all three copies of the “a” sequence of HSV-1(KOS).

h The six clusters of genes or exons with unusually low GC content are shown in bold.

i Abbreviations: TR, tandem repeat; N-term, N terminal; dom, domain; memb, membrane; C term, C terminal; esp, especially; cons, conservation; SS, single stranded; UDG, uracil DNA glycosylase; Frag, fragmented; F, forward strand; C, complementary strand.

The gene-ORF-protein nomenclature used here for EEHV4(Baylor) is also based on that used originally for EEHV1B(Kiba) by Ehlers et al. (17) and expanded upon for EEHV1A(Kimba) by Ling et al. (24) to include an E-series numbering system for all novel *Proboscivirus*-specific proteins. All proteins with identifiable orthologues in EEHV1A(Kimba) retain those same numbers, whereas all newly assigned proteins that are unique to EEHV4 have been given distinctive E number descriptors.

**FIG 2:**
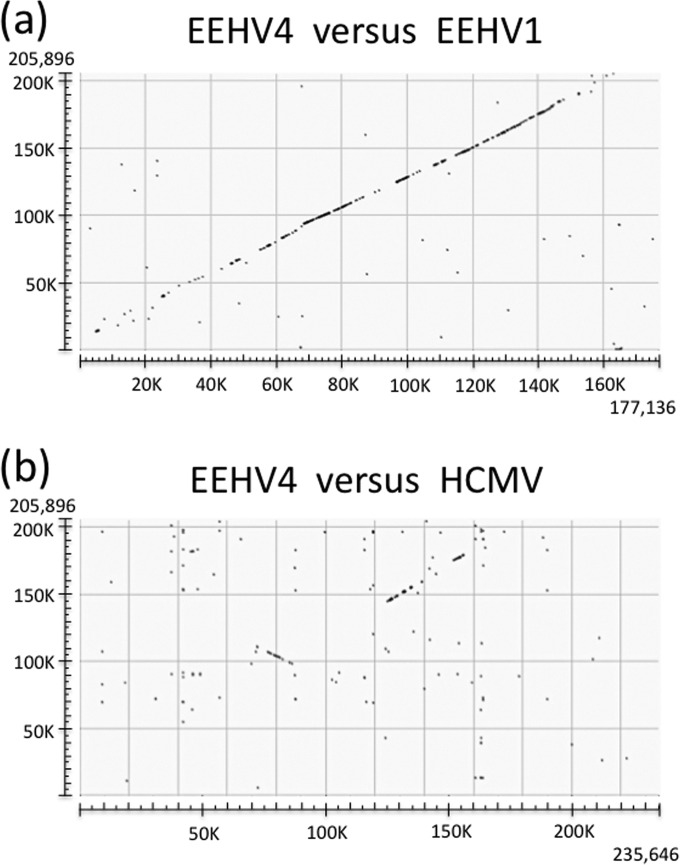
Global alignment patterns for the intact EEHV4 genome compared to EEHV1 and HCMV. The dot matrix diagrams showing direct linear nucleotide alignments were generated as implemented at http://blast.ncbi.nlm.nih.gov/Blast.cgi. (a) Comparison across the intact 206-kb genome of EEHV4(Baylor) (KT832477) from the GC-rich branch of the *Proboscivirus* genus with the intact 180-kb genome of EEHV1A(Kimba) (KC618527) from the AT-rich branch of the *Proboscivirus* genus derived from the work of Ling et al. (24) when aligned in the same orientation. (b) Comparison across the intact 206-kb genome of EEHV4(Baylor) (KT832477) with the intact 235-kb genome of HCMV(Merlin) (AY446834.2) in the *Cytomegalovirus* genus of the mammalian betaherpesvirus subfamily derived from the work of Dolan et al. (41), with the latter aligned in the standard orientation.

The most obvious feature about the EEHV4 genome is that the overall orientation and gene content (especially within the central core gene segment) are essentially conserved and colinear with those of the three AT-rich branch genomes, although with considerable divergence toward both ends as revealed in a full-length genomic dot matrix comparison (Fig. 2a). In particular, the large 40-kb inversion of the conserved core blocks I, II, and III between U27 and U44 in betaherpesviruses and a second smaller inversion of a weakly conserved 24-kb gene block are both retained in common with the organization found in EEHV1A, EEHV1B, and EEHV5. Part of the core gene region involved in the first of these two genomic inversions is clearly revealed on the left side within a dot matrix comparison with the prototype betaherpesvirus genome human cytomegalovirus Merlin [HCMV(Merlin)] (Fig. 2b), although the second inverted region (further toward the left side) is not sufficiently conserved to be detectable in the diagram. The other half of the conserved core segment (blocks IV, V, VI, and VII) produces the largest visible signal, located further toward the right side, but is not inverted. No other mammalian herpesviruses have this same type of overall gene organization as found within both major branches of the EEHVs.

In common with the pattern found for the other *Proboscivirus* genomes, the central core segment of EEHV4 (Fig. 1) retains the three signature optional shared alpha-gamma (αγ) and alpha-beta2 (αβ2) class genes U27.5 (RRB), U48.5 (TK), and U73 (origin-binding protein [OBP]), plus two other betaherpesvirus-like genes, U47 (gO) and U51 (vGPCR1), mapping together with the 35 obligatory true core genes found in all mammalian herpesviruses, and the six other shared beta-gamma (βγ) class genes that are absent from all alphaherpesviruses. Although 15 other genes in the HCMV US22, vMIP, vICA, and mIE gene block from UL23 through UL43 are clearly absent and the whole 24-kb genome segment from E32 (U14.5) to E35 in EEHV4(Baylor) at coordinates 60.5 to 81.6 lies in an inverted orientation compared to that of the adjacent blocks in all betaherpesvirus genomes, some of the genes designated U14.5, U14, U13.5, U12 (vGPCR2), U4, and U4.5 (ORF-B) in EEHV1A(Kimba) have low-level resemblance to a subset of their betaherpesvirus orthologues in this region. Because of the evidently shared common evolutionary origin within a predicted ancestor of both subfamilies, these six genes in both EEHV1A and EEHV4 have been assigned beta-*Proboscivirus* (β*p*) or beta-delta (βδ) class status (24) (Table 1).

**FIG 3:**
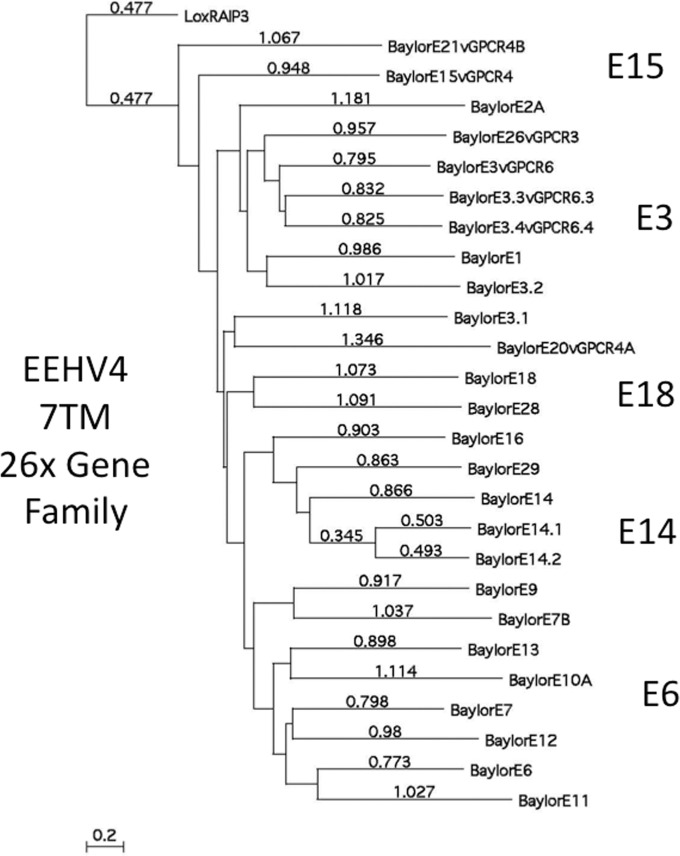
EEHV4 carries a very large family of distantly related paralogous 7xTM and vGPCR-like genes. Linear distance-based Bayesian bootstrap phylogenetic tree comparisons for all 26 members of the 7xTM-containing multigene family from the EEHV4(Baylor) GC-rich branch *Proboscivirus* compared to their nearest host cell analogue Lox RAIP3 as the outgroup. The entire family is loosely divided into five subgroups, whose designated prototypes of E15, E3, E18, E14, and E6 are indicated. Note that, as indicated by the distance values, all of these paralogues are very highly diverged from one another, with the exception of E14.1 and E14.2, which most likely represent the most recent duplication event. A subset of the genes in this family (*p*3 or δ3) exhibit features of GPCR genes as described in greater detail in the accompanying paper (27).

A dramatic feature of the EEHV4(Baylor) genome is the presence of 26 paralogous members of an ancient and highly diverged family of 7xTM anchored transmembrane proteins (designated the *p*3 or δ3 family) within the left-side novel segment of the genome. The linear distance-based protein level phylogenetic tree given in Fig. 3 shows relationships and subgrouping among this protein family, of which the closest viral or cellular homologue is retinoic acid-induced protein 3 (RAIP3), an orphan group G-protein-coupled receptor (GPCR). Although they are very highly diverged, a combination of the phylogenetic tree branching patterns together with PBLAST domain-match identity values (not shown) allowed us to loosely classify them into five subfamilies, based on closer relationships to the prototype examples of E3, E6, E14, E15, and E18, of which there are seven (E1, E3, E3.1, E3.1, E3.3, E3.4, and E26), six (E6, E7, E9, E11, E12, and E13), four (E14, E14.1, E14.2, and E16), three (E15, E20, and E21) and two (E18 and E28) paralogous copies, respectively, in EEHV4. Only the adjacent pair E14.1 and E14.2 display a much closer identity than any others, suggesting that they are the most recently duplicated and most likely both arose from the adjacent gene, E14. More detailed descriptions and comparisons of the multiple vGPCR-like proteins as well as several other smaller gene families and some captured cellular genes from both major branches of the *Proboscivirus* genus and related mammalian herpesvirus proteins are presented in the accompanying manuscript by Ling et al. (27).

Another striking feature of the EEHV4(Baylor) genome assembly is the evident absence of the entire 10.5-kb block containing the 10 to 12 highly variable genes mapping between E47 and E55 of EEHV1A(Kimba) or of the rearranged versions of this block found in EEHV1B(Emelia) and EEHV5(Vijay). Those genes include between three and five members of the CD48-related vIgFam gene family plus several vGPCRs, as well as the E47 (vFUT9) and E54 (vOX2-1) genes. All six copies of the signature 10- to 35-bp-long alternating CA repeats found in EEHV1A(Kimba) are also missing in EEHV4(Baylor), although that was already known to be the case for EEHV5(Vijay) as well.

Although the intact genomes of EEHV4(Baylor) and EEHV1A(Kimba) are too far diverged for accurate global determinations of the overall DNA sequence identity level between them, a numerical value of 49.6% was measured by the Emboss DNA Stretcher program for the most conserved 97-kb core genomic segment from U44 (position 82500) to U77 (position 179500). Distortions to the linearity of the alignments preclude obtaining meaningful results for the other noncore outer parts of the EEHV4 genome. This result compares to values of 63% identity for the intact EEHV1B(Emelia) genome versus EEHV5(Vijay) and 92.3% for EEHV1B(Emelia) versus EEHV1A(Raman), which increase to 64.2% and 94.2%, respectively, when the equivalents of the 10.5-kb nonlinear vIgFam-plus-vGPCR block between E47 and E55 in Kimba are omitted.

### Unusual patterns and distribution of GC-rich sequence blocks and A or T tracts.

The several small previously analyzed PCR segments of EEHV3 and EEHV4 DNA (totaling 4 to 5 kb each) were recognized to have a distinctive much-higher overall GC content of 63% compared to 43% for EEHV1A, -1B, and -5 (11, 20). However, the overall base composition of the intact EEHV4 genomic DNA proved to be just 57% GC content. Much of this difference results from the curious finding that, unlike the adjacent protein coding regions, the majority of the interspersed non-protein-coding regions in EEHV4 are highly conspicuous by the inclusion of numerous successive A or T tracts of between 5 and 11 nucleotides in length. These features also apply within the relatively small number of clearly identifiable introns.

Despite the lower-than-expected overall GC content, the level does increase to an average of 62% (range, 57 to 66%) for the 17 largest protein coding regions, which are all over 2,500 bp in length and occupy 67.6 kb. In fact, all but 15 of the 119 recognized ORFs display a GC content of greater than 48% (Table 1), whether they map within the novel left part of the genome or within the core conserved region. The only exceptions to this pattern occur across six small dispersed locations (boldface in Table 1) encompassing just 14 kb in total length and containing genes that are either very highly diverged from their counterparts in EEHV1 and EEHV5 or entirely novel. Five of these genes, including the additional second captured acetylglucosamine transferase E9A (vOGT), lie adjacent to one another between map coordinates 20.8 and 26.1 kb. The second largest such locus covers the E37 (ORF-O) and adjacent novel E39A (ORF-R) glycoprotein between map coordinates 186 and 189.7. The third locus contains two genes, including a captured E16D (vECTL) protein mapping between map coordinates 35.6 and 36.8. Finally, E4A at coordinates 15.2 to 15.7 and E30A mapping between coordinates 55.5 and 56.2 also fall into this category. Perhaps reflecting a relatively more recent acquisition of at least one of the genes at each of these five AT-rich ORF locations, they appear to be much more similar to host cell DNA than to the rest of the viral genome, with ORF base compositions ranging from only 27 to 45% GC content.

### Novel EEHV4 type-specific genes.

The currently updated annotation of the EEHV1A(Kimba) genome contains 118 identified genes. Among these, a total of 26 are evidently missing within the EEHV4(Baylor) genome), whereas a total of 25 additional apparently novel genes are present (Table 1). The missing genes in EEHV4(Baylor) relative to EEHV1(Kimba) include, in addition to the entire 10.5-kb 10-gene block from E47 to E55, those designated E2, E5A, E7A, E8, E10, E16A/B, E17, E18A, E18B, E24, E25, E31, E36A (vCXCL1), E38 (ORF-P), and E39 (ORF-Q). The extra genes in EEHV4(Baylor) compared to EEHV1A(Kimba) include E3.1, E3.2, E2A, E3.3, E3.4, E4A, E4B, E4C, E6B, E7B, E9A (vOGT), E9B, E9C, E10A, E12A, E14.1, E14.2, E16D (vECTL), E17A, E18C, E20B, E24B, E27ex2, E30Aex1/2, and E39A (ORF-R). There are also two genes, EE23A (vOX2-V) and EE44A, that are unique to EEHV5(Vijay) and that are absent from both EEHV1 and EEHV4, and E39 (ORF-Q) is unique to EEHV1A and EEHV1B but absent from EEHV2, EEHV4, and EEHV5, whereas E33A and E36A (vCXCL1) are unique to EEHV1A although absent from EEHV1B, EEHV2, EEHV4, and EEHV5.

Six of the new genes in EEHV4 represent recognizable highly diverged duplicated paralogs of genes already present in both EEHV1 and EEHV4 that could presumably have been deleted from some ancestral genome as the AT-rich branch evolved separately from the GC-rich branch. All of the latter are members of the *p*3 (or δ3) 7xTM family described above (E3.1, E3.2, E3.3, E3.4, and E14.1 and E14.2). E2A, which maps among the new duplicated versions of the vGPCR6 cluster, is also clearly a member of this 7xTM family, but this protein has no recognizable orthologues or paralogues in either EEHV1 or EEHV5. A similar situation applies for E7A and E10A. In contrast, the following remaining 16 members of the 7xTM family, E1, E3, E7, E9, E11, E12, E13, E14, E15, E16, E18, E20, E21, E26, E28, and E29, all have direct orthologues in EEHV4 as recognized by both positional and protein homology criteria.

EEHV4 E37 (ORF-O) and E39A (ORF-R) are both predicted to be spliced potential envelope glycoproteins mapping between gL and ORF-K at coordinates 186 to 189.6 at the same location and orientation as the similarly spliced E37 (ORF-O) plus E38 (ORF-P) and E39 (ORF-Q) Ser-plus-Thr-rich glycoproteins of EEHV1A(Kimba). However, although quite plausibly these proteins all had common evolutionary origins, ORF-R now lacks sufficient residual homology to be designated an unambiguous orthologue of either ORF-P or ORF-Q. Interestingly, all three proteins have two exons, and residual identity between ORF-P and ORF-Q (especially within exon 1) suggests that ORF-Q is a highly diverged tandem-duplicated version of ORF-P that is present only in EEHV1A and EEHV6 and is missing from EEHV1B, EEHV2, and EEHV5. However, both ORF-P and ORF-Q are absent from EEHV4 and have seemingly been replaced by (or evolved into) the much shorter ORF-R glycoprotein that now displays no detectable identity to either of them. In contrast, the adjacent ORF-O glycoprotein of EEHV4, predicted to be three exons, still retains significant identity (37% across all of exon 3, although barely detectable in exons 1 and 2) to its orthologues in all of the AT-rich probosciviruses.

### Various patterns of base composition bias within wobble codon positions.

We previously pointed out that the coding regions within each of the four to five small segments sequenced by Sanger PCR approaches from both prototype genomes, EEHV3(NAP27) and EEHV4(NAP22), within the GC-rich branch of the probosciviruses displayed extraordinarily high GC content bias at the third nucleotide or wobble codon position (11). For five of the seven short ORF fragments analyzed there in each virus, the wobble position GC content was between 86 and 99%. This compares with codon wobble position GC contents ranging from just 41 to 50% for the orthologous ORF segments in EEHV1 and EEHV5.

**FIG 4:**
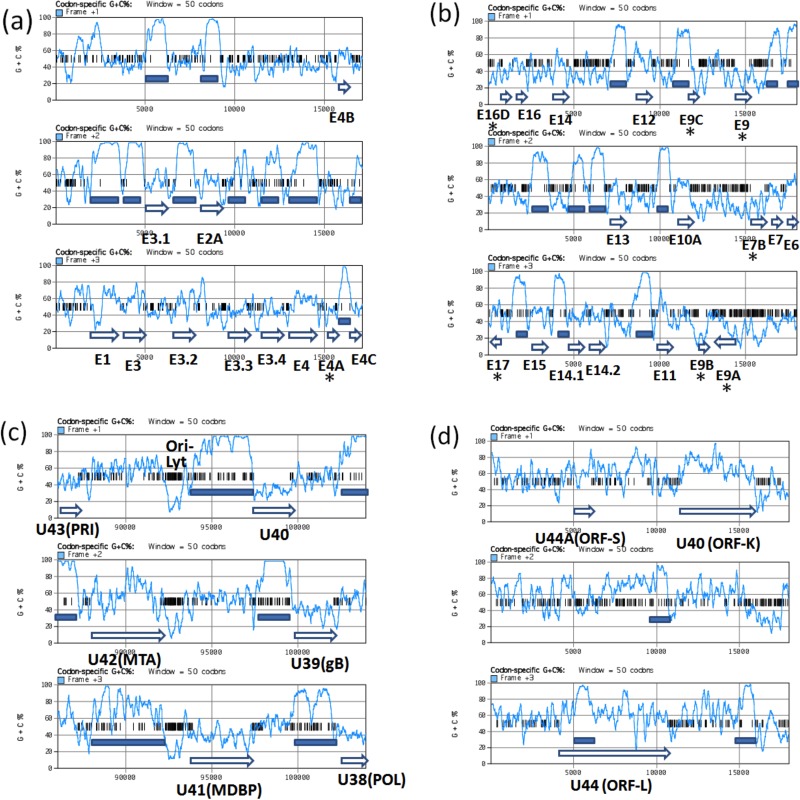
Codon-specific scanning GC content panels showing the wobble codon GC bias effect across selected representative segments of the EEHV4(Baylor) genome. Diagrams showing the percent G-plus-C content of each of the three potential translated codon frames across four selected 18-kb segments of the EEHV4B(Baylor) genomic DNA sequence as implemented under the codon-specific G-plus-C percent toolbox item in MacVector 12. Short vertical bars indicate forward direction terminators. Annotated ORF positions and sizes are denoted by open arrows. Highly GC-biased wobble position blocks with average values between 80 and 100% are marked with solid bars. For a hypothetical ORF with an initiator codon beginning in frame 1 at position *x* in the diagram, the wobble position codons are represented by the succeeding frame 3 line (or frame 1 for a frame 2 initiator and frame 2 for a frame 3 initiator). (a) Forward-directed strand across coordinates 1 to 18000 at the extreme left side encompassing 10 out of 11 rightward-oriented genes from E1 to E4C, including E4 (vGCNT1) with high wobble position GC bias. (b) Inverted segment of the complementary strand across coordinates 37000 to 19000 encompassing predominantly leftward-oriented genes (16 between E6 and E16D) and two rightward-oriented genes (E9A and E17), including 11 genes displaying uniformly high wobble codon GC bias plus seven genes in two blocks, E7B-E9 (vOGT)-E9A-E9B-E9C and E16D (vECTL)-E17-E17A, that do not display wobble GC bias (all of the latter are labeled with asterisks). (c) Forward strand across coordinates 86000 to 104000 encompassing six rightward-oriented core region genes, U43 (PRI) to U38 (POL), with high wobble position GC bias on either side of the predicted novel Ori-Lyt domain. (d) Inverted segment of the complementary strand from the extreme right side across coordinates 187867 to 205894 encompassing U44A (ORF-S), U44 (ORF-L), and U40 (ORF-K). The only three high-GC-bias wobble codon blocks found within this region occur in ORF-S and in the conserved C-terminal domains of ORF-L and ORF-K (marked with solid bars).

Evaluation of the complete 206-kb EEHV4(Baylor) genome shows that a very high level of GC content bias at the third nucleotide or wobble codon position applies across close to 90% of the entire genome, involving as many as 105 of the 119 total annotated genes. Remarkably, a global plot comparison of the GC contents of each of the three EEHV4 reading frames as an indirect measure of the wobble position effect produces such a dramatic pattern that the position of virtually every ORF (except those in the six minority AT-rich regions) is easily recognized and defined. However, there is very little similar frame-specific demarcation when this type of plot is applied to EEHV1A(Kimba). Four selected examples of these codon-specific plot panels scanning 18-kb segments each of the EEHV4(Baylor) genome are shown in Fig. 4a to d, two from novel areas of the genome on the left side, one showing a conserved core gene segment surrounding Ori-Lyt near the center of the genome, and one encompassing the presumed transcriptional regulators ORF-K and ORF-L at the extreme right side. Typical examples of large genes with a high GC wobble bias in EEHV4(Baylor) include U57, encoding the major capsid protein (MCP), plus U41 encoding the major single-stranded DNA-binding protein (MDBP or SSP) (Fig. 4c) and also the novel EEHV-specific gene E4, encoding the captured vGCNT1 protein (Fig. 4a). These proved to have wobble position GC contents compared to overall ORF GC-contents of 96.6% (63% aa identity), 89% (65%), and 86% (61%), respectively. Omitting 318 amino acids (aa) at the ends of the U57 protein (MCP) from the analysis leaves a central 3,000-bp segment with an extreme wobble position GC content of 99.5%. The other two proteins also have a typical tendency toward deviations from the wobble position GC bias near one or both ends. Almost all of the well-conserved common herpesvirus core proteins, as well as a subset of the more novel and diverged group-specific proteins in EEHV4, also follow this trend.

The major exceptions to this very high wobble position GC content bias pattern include all 14 genes mentioned above that map within the six small well-dispersed loci with 48% or less overall GC content (boldface in Table 1). Ten of these genes display wobble position GC contents very close to their overall GC content as follows: E4A, 46% (44%); E7B, 29% (27%); E9, 31% (27%); E9A (vOGT), 46% (42%); E9B, 26% (23%); E9C, 25% (27%); E16D (vECTL), 45% (45%); E17, 53% (45%); E30A, 39% (42%); E37 (ORF-O), 37% (41%). In contrast, the E39A (ORF-R) glycoprotein shows an apparently enhanced bias in the opposite direction (i.e., toward even lower GC richness) of 26% wobble GC content compared to 41% overall GC content. The first of those aberrant genes is included in the three-frame GC scan diagram shown in Fig. 4a, and the next seven are shown in Fig. 4b.

Interestingly, although 11 genes that do not follow the typical EEHV4 pattern of high GC bias neither represent core genes nor are shared with any other virus groups, they do include a curious mixture of both obvious novel captured cell genes (vOGT and vECTL), plus the unique ORF-R glycoprotein and several other novel genes that are also not shared with EEHV1 and EEHV5 (E4A, E7B, E9C, and E30A), together with some adjacent but highly diverged genes that are shared (E9, E9B, E17, and ORF-O). They even include two apparent members of the *p*3 or δ3 7xTM multigene family (E7B and E9), although the first of these has no direct matching identity to any other EEHV4 7xTM protein. Therefore, although some of the AT-rich genes may indeed be relatively newly captured cellular genes, it would be hard to argue that they were all acquired by any kind of common mechanism or event.

The two adjacent large 1,465-aa and 2,065-aa leftward-oriented potential transcriptional regulatory proteins E40 (ORF-K) and E44 (ORF-L) of EEHV4 are especially intriguing in this regard with unusual mixed bias patterns (Fig. 4d, inverted orientation). Both have a total wobble position GC content of just 69% or 63%, matching closely their overall GC contents of 66% and 64%, respectively. However, this is not distributed uniformly, with just small 230-aa and 244-aa C-terminal domains in both (matching the segments that are conserved within the otherwise highly diverged EEHV1 and EEHV5 orthologues) having wobble position GC contents of 86% and 90%, respectively. In contrast, another segment of ORF-L that overlaps a potential second much-smaller 296-aa coding region within a different reading frame here (26), known as EE1A, E44A, or ORF-S, has a wobble position GC content of just 43%. However, this novel internal overlapping ORF-S protein of EEHV4 itself (which is conserved at the 35% identity level in EEHV1 and EEHV5) has a typical wobble position GC content of 89% (overall, 65%). Unexpectedly, in contrast to the situation in the AT-rich branch probosciviruses EEHV1A(Kimba), EEHV1A(Raman), EEHV1B(Emelia), and EEHV5(Vijay) (18, 25), where the entire ORF-L coding region and adjacent upstream region display highly localized CpG suppression similar to that seen in the MIE1 (UL123) gene region of cytomegalovirus and many other betaherpesviruses (28), this feature is completely absent from the EEHV4 version of ORF-L.

### Complex enlarged Ori-Lyt dyad symmetry domain.

**FIG 5:**
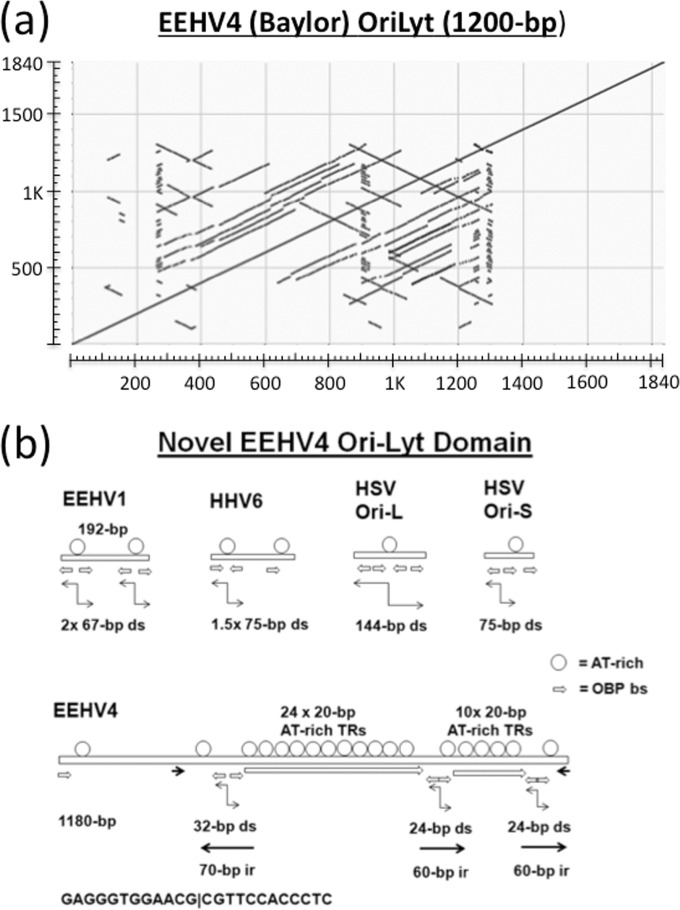
Complex tandem and inverted repeat patterns within the predicted Ori-Lyt domain of EEHV4. (a) Dot matrix self-comparison of the DNA from coordinates 92021 to 93860 encompassing the entire intergenic region between the N terminus of U41 (MDBP) and the C terminus of U42 (MTA) from EEHV4(Baylor). The proposed 1.2-kb Ori-Lyt domain spans from coordinates 92120 to 93325. Direct tandemly repeated structure is indicated by additional lines parallel to the main diagonal, whereas inverted repeats are indicated by additional lines perpendicular to the main diagonal. (b) Features of the unusual expanded dyad symmetry Ori-Lyt region of EEHV4 compared to those of EEHV1 and other alphaherpesvirus-like dyad-symmetry-type origins. Cartoon diagram comparing the sizes and major structural features of the predicted dyad symmetry domains of EEHV4(Baylor) and EEHV1A(Kimba) with those of the HHV6 version and with both Ori-L and Ori-S of HSV-1. Circles denote alternating A-plus-T dinucleotide runs. Short horizontal pointed bars represent copies of the consensus OBP-binding site motif. Other, larger sets of arrows designate various types of inverted repeats as well as the 24× and 10× copies of the 20-bp AT-rich direct tandem repeats in the EEHV4(Baylor) version.

Similar to the other EEHVs, EEHV4 encodes a U73 orthologue of the alphaherpesvirus herpes simplex virus (HSV) UL09 type of origin-binding protein (OBP), but the expected matching dyad symmetry Ori-Lyt region mapping between the U41 (MDBP) and U42 (MTA) genes of EEHV4 is much larger and more complex than those of EEHV1 and EEHV5 and was initially very difficult to sequence because of unusual features attributed to several internal stem-loop structures. The entire combined inverted and direct repeat arrangement encompassing the predicted EEHV4A(Baylor) 1,180-kb Ori-Lyt domain from map coordinates 92120 to 93324 is illustrated in the dot matrix self-comparison plot presented in Fig. 5a. A cartoon representation of the structure is also shown in Fig. 5b for comparison with the only 75- and 192-bp versions from HSV Ori-S, HSV Ori-L, human herpesvirus 6 (HHV6) Ori-Lyt, and EEHV1/5 Ori-Lyt. The EEHV4 locus has a total of seven copies of the consensus OBP-binding site (OBS) sequences (GAG)GGTGGAACG present compared to just three to four in the other viruses. Four of these OBS motifs in EEHV4 are arranged as pairs of direct head-to-head palindromic copies, whereas in the other viruses (as well as in a third pair in EEHV4), they mostly have 5- to 7-bp gaps between the head-to-head binding site pairs.

Most of the other herpesvirus Ori-Lyt regions of this type (18, 24, 25, 29) have a single central very AT-rich loop between the inverted repeated stems containing the OBP-binding sites (although this is duplicated in EEHV1 and EEHV5 compared to HHV6), but the EEHV4 version instead consists predominantly of two large loops with multimerized copies of 20- to 22-bp-long AT-rich direct tandem-repeat elements bounded by three nearly identical 60- to 70-bp inverted repeats of the binding site containing stem-loops. There is also an additional pair of 40-bp inverted repeats encompassing this whole 800-bp block, with a few additional repeated elements lying within an apparent degenerate area beyond that on the left side.

### Clustered palindromic CREB-binding site motifs.

A very characteristic feature of primate cytomegaloviruses is the large and complex major immediate-early (MIE) enhancer domain mapping directly upstream from the genes encoding the spliced overlapping MIE1 (UL121) and MIE2 (UL123) transcriptional regulatory proteins. In particular, the 750-bp core HCMV MIE enhancer contains several sets of dispersed multimerized *cis*-acting elements encompassing consensus binding sites for the cellular transcription factors AP1, NF-κB, and CREB located upstream of the TATATAA box motif. In particular, there are eight copies of the very-high-affinity doubled-up 8-bp palindromic versions of cyclic AMP (cAMP) response elements (CREs) that have in isolation been shown to provide both powerful basal transcriptional effects and responsiveness to cAMP, Ca^2+^-forskolin, and tetradecanoyl phorbol acetate (TPA) (30, 31). These same multimerized *cis*-acting elements are also present in rearranged patterns within African green monkey (AGM), rhesus, and baboon CMV MIE enhancers, together with additional accessory control elements, including tandemly repeated further-upstream NF1-binding site clusters and in some cases a large stretch of bent DNA (32–34). Well-characterized repetitive control elements of this type are not present upstream of the equivalent MIE genes of muromegaloviruses or roseoloviruses; therefore, it was surprising to find a closely spaced cluster of both consensus 8-bp (TGACGTCA) and lower-affinity 5-bp (TGACG) half-site CRE motifs within the terminal repeat regions in all three of the EEHV1, EEHV5, and EEHV4 genomes (Table 1). In EEHV1, there are six copies of the palindromic 8-bp CREs within a 153-bp segment and eight within an extended 590-bp segment but no others at all within the rest of the entire 180-kb genome, whereas in EEHV4, while four of the total of six 8-bp CREs occur within this cluster, there are also six more of the 5-bp CREs within the same 540-bp block. By stochastic chance, a single 8-bp CRE motif should occur just once every 64,000 bp of average-GC-content DNA, and a second one within the same 540-bp region would occur about 150-fold less frequently. Therefore, these are hardly random occurrences. Intriguingly, in the circularized form of the EEHV genomes the CRE clusters would all lie within a large noncoding region between 2.5 kb and 6.5 kb directly upstream of the candidate E44 (ORF-L) transcriptional transactivator protein gene at one end, as well as in EEHV1 and EEHV5 only just a few hundred base pairs upstream from the E47 (vFUT9) gene at the other end of the genome. A pair of these same 8-bp CRE motifs are also key functional elements in the long terminal repeat (LTR) enhancers of both human T-cell leukemia virus type 1 (HTLV-1) and HTLV-2. Therefore, based on the astronomical odds against them occurring by chance, together with the apparent strategic location, it seems reasonable to speculate that these clusters could play some important role either in packaging events or in transcriptional regulation in the EEHVs.

### Chimeric subtype-level divergence patterns from the prototype EEHV4(NAP22) genome.

To address whether or not there may be chimeric domains of the CD-I, CD-II, and CD-III type described previously that distinguish EEHV1A from EEHV1B (18) and EEHV5A from EEHV5B (11, 13), selectively targeted PCR loci totaling 24.3 kb were Sanger sequenced from necropsy heart tissue DNA of the prototype EEHV4(NAP22) genome in addition to the 5.7 kb already available from that strain (1, 10, 11). While most loci showed minimal differences at the nucleotide level (0.3 to 2.5%), three gene blocks encompassing glycoprotein U39 (gB) plus U46 (gN)-U47 (gO)-U48 (gH) as well as the O-linked acetylglucosamine transferase E9A (vOGT) instead proved to be highly diverged. The results across a contiguous 4.9-kb segment, including gN to gH, revealed a clearly defined contiguous chimeric domain of nearly 3.7 kb at Baylor equivalent coordinates 131750 to 135400, which almost exactly matches the same position and size as CD-II of EEHV1B compared to EEHV1A (18). This complete CD-II-like block displayed 26.3% total nucleotide divergence, with the individual protein-level (and DNA-level) differences being 9% (11%), 23% (31%), 32% (37%), 26% (27%), and 13% (13%), respectively, for the intact E35A (ORF-J), U46 (gN), U47 (gO), U48 gH), and nearly intact U48.5 (TK) genes. For the 1.6-kb segment of the U39 (gB) gene evaluated, the results for the EEHV4(NAP22) version revealed a much smaller version of CD-I than in EEHV1 with 189 nucleotide polymorphisms over an internal 1,150-bp segment mapping between EEHV4(Baylor) coordinates 99993 and 101150 representing 16.4% DNA-level and 14% amino acid divergence with sharp chimeric boundaries on both sides. Similarly, because we could not amplify any of the expected gene block between UDG and the C terminus of ORF-K for EEHV4(NAP22) using EEHV4(Baylor)-based primers, we deduce that the latter region encompassing UDG, gL, ORF-O, and ORF-R most likely represents a highly diverged chimeric domain of a similar size and location as CD-III of EEHV1. Finally, a 6.6-kb sequenced block surrounding the novel captured vOGT gene proved to encompass a new fourth 4,660-bp chimeric domain designated CD-IV with sharp chimeric boundaries mapping at coordinates 20541 to 25200. This domain encompasses six genes, including part of E7 plus all of E7B, E9, E9A (vOGT), E9B, and E9C and displays 741-bp (16.0%) overall nucleotide polymorphism. The five intact proteins involved diverge by 28%, 22%, 13%, 18%, and 28%, respectively, whereas the values for the adjacent E7 ORF, all of E10A, and part of E11 analyzed are instead just 3%, 2%, and 0%, respectively. Intriguingly, in addition, the E9C and E9B ORFs have become fused in frame in EEHV4A(NAP22) rather than occupying two different reading frames as in EEHV4B(Baylor).

**FIG 6:**
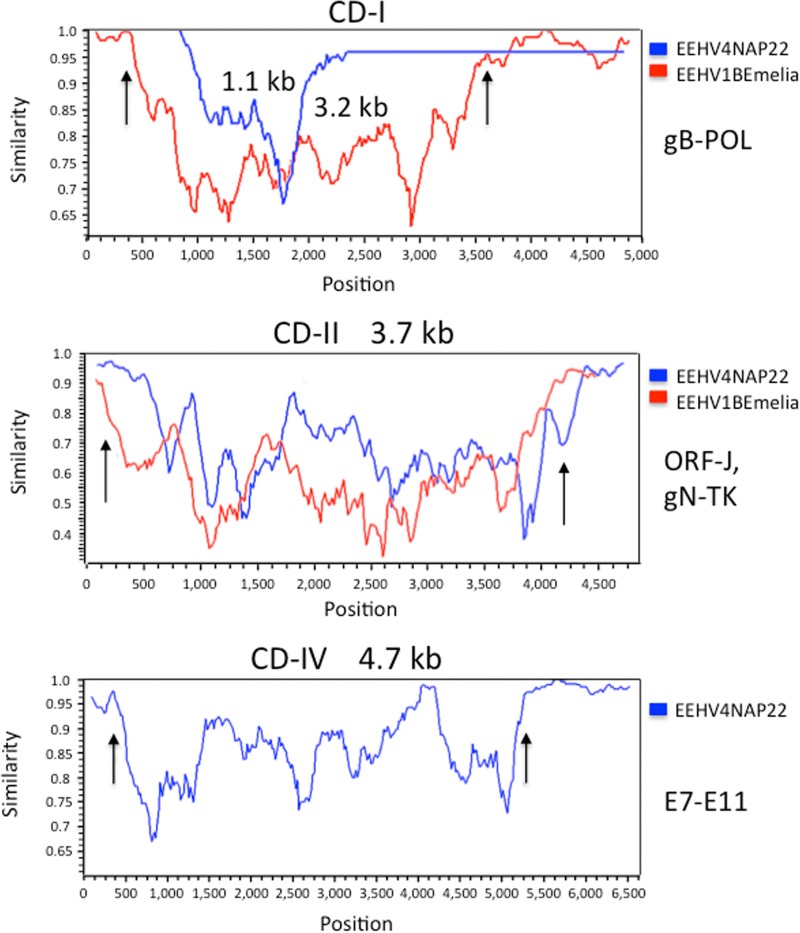
Positions and sizes of three identified EEHV4A-EEHV4B chimeric domains and boundaries relative to those of EEHV1A-EEHV1B chimeric domains. The diagrams show Simplot (40) comparisons of the nucleotide identity patterns between EEHV4A(NAP22) and EEHV4B(Baylor) across three mapped chimeric domains, CD-I (1.1 kb), CD-II (3.7 kb), and CD-IV (4.7 kb), shown as blue lines in comparison to superimposed data for CD-I (3.2 kb) and CD-II (3.7 kb) of EEHV1A(Kimba) versus EEHV1B(Emelia), shown as red lines. CD-IV of EEHV4 has no equivalent in EEHV1, and there are no data available for the presumed region CD-III of EEHV4. (Top) CD-I chimeric region within U39 (gB) of EEHV4A versus EEHV4B at EEHV4(Baylor) map coordinates 99993 to 101150 compared to the much larger overlapping CD-II of EEHV1A versus EEHV1B, which encompasses part of U40, all of U39 (gB), and part of U38 (POL)**.** Vertical arrows denote the positions of the EEHV1A1B chimeric domain boundaries. (Middle) CD-II chimeric region encompassing part of ORF-J, all of gN-gO-gH, and part of TK of EEHV4A versus EEHV4B at EEHV4(Baylor) map coordinates 131750 to 135400 compared to the nearly equivalent superimposed CD-II of EEHV1A versus EEHV1B. Vertical arrows denote the positions of the EEHV1A-1B chimeric domain boundaries. (Bottom) CD-IV chimeric domain of EEHV4A versus EEHV4B mapping between EEHV4(Baylor) coordinates at map coordinates 20541 to 25210. Vertical arrows denote the positions of the EEHV4A-4B chimeric domain boundaries that encompass part of E7 and all of E7B, E9, E9A (vOGT), E9B, and E9C but end before E10A.

A pictorial illustration of the high (species-level) patterns of divergence between these first two known examples of EEHV4 strains within the equivalents of the CD-I and CD-II chimeric blocks are presented in the Simplot comparisons given in Fig. 6, top and middle. These diagrams include matching to-scale comparisons with the equivalent CD-I and CD-II chimeric blocks of EEHV1B(Emelia) versus EEHV1A(Kimba) as described by Richman et al. (18). The apparent new CD-IV chimeric domain in EEHV4 across the E7-to-E9C gene block and including the novel captured E9A (vOGT) gene is also shown as a Simplot comparison in Fig. 6, bottom, but there is no known equivalent at this position in EEHV1A-1B or in EEHV5A-5B. In addition, an intermediate level of nucleotide variability of 11 to 12% was found at three other smaller loci for E4A, E31B, and U50-U51 (see Table S1 in the supplemental material), although 40% of the latter occurs within noncoding regions, whereas for the vGPCR1 ORF itself the DNA and protein variability levels reach only 7.8% and 6.7%, respectively. Some additional EEHV4(NAP22) PCR sequencing was also carried out to confirm predicted ORFs and consensus splicing patterns at loci such as E4A, E4B, E4C, E6B, E12A, E16D (vECTL), E17, E17A, E18C, E20B, E23B, and E31B that were somewhat ambiguous from the EEHV(Baylor) data alone. Overall, it seems amply justified to refer to EEHV4(NAP22) as the prototype example of an EEHV4A subtype, whereas EEHV4(Baylor) would be the prototype of an EEHV4B subtype. The total of 30 kb of DNA sequence now available for EEHV4(NAP22) represents nearly 15% of the total expected genome size and gives an overall measured strain divergence from EEHV4B(Baylor) of 8.8% (2,684-bp polymorphisms [see Table S1]). However, that value drops to just 1.9% when the 8.4 kb of data from the three observed CD-like chimeric domains and the variable vGPCR1 locus are omitted. Finally, it is also very evident when comparing the two chimeric subtypes of EEHV4 that the intergenic domains represent rapidly changing sequences.

10.1128/mSphere.00081-15.1 Table S1  Detailed differences between PCR-sequenced regions of EEHV4A(NAP22) and EEHV4B(Baylor). Download Table S1, PDF file, 0.1 MB. Copyright © 2016 Ling et al. 2016 Ling et al. This content is distributed under the terms of the Creative Commons Attribution 4.0 International license.

### Accuracy of the predicted splicing patterns.

Of necessity, because of the inability to grow EEHVs in cell culture, all predicted splicing patterns in EEHV genes are provisional. However, there are multiple levels of expected accuracy involved. All of our predictions are based on finding typical patterns of herpesvirus splicing involving strong to medium consensus donor and acceptor sites, generally small 60- to 150-bp introns, and the need to jump across frameshifts in most of the introns to generate a logical intact protein. In addition, for EEHV1 we had relied on consistent conserved splicing signal locations and patterns across numerous distinct strains with sometimes otherwise quite variable sequences. For four of the predicted spliced proteins of EEHV4, namely, U12 (vGPCR2ex1,2), U60-66 (TERex1,2,3), U42 (MTAex1,2), and U37 (ORF-Oex1,2,3), not only do the consensus motifs and patterns match those in multiple strains of EEHV1A (18, 24), but they each also agree with the predictions made by at least one other group for EEHV1A, EEHV1B, or EEHV5 (17, 25, 26). The U12 pattern also matches that found in HCMV, and the U42 (MTA) pattern does so for the EBV orthologue. For EEHV4 ORF-Rex1,2, although it lacks homology with the positional equivalents ORF-P and ORF-Q of EEHV1A, EEHV1B, or EEHV5, the predicted splicing is consistent with the similar interpretations for them by both our group (18, 24) and the group of Wilkie et al. (25, 26). Several other genes, including E17 and E27, also match our predicted splicing patterns from multiple strains of the EEHV1 versions. However, the situation for EEHV4 E24B (vOX2-B), just as for the highly diverged equivalents in EEHV1A, EEHV1B, and EEHV5, is much more complex and speculative. In all three of the latter, the authors of the complete genome papers made logical predictions of multiple spliced exons and proteins, which are conserved across many highly diverged strains (and even include a partial match to a host OX2 splicing motif), but they have no predictive value for the EEHV4 version. The simplest plausible interpretation that we could make was the presence here of two partially overlapping proteins, namely, E24Bex1,2 and E23B, of which only the former has amino acid identity to viral and cellular OX2 proteins. However, the presence of multiple additional consensus splicing signals here could also be interpreted to generate four separate alternatively spliced proteins, including one that joins parts of both E24B and E23B from a total of six different exons plus four donor sites and four acceptor sites mapping in frame across the region between positions 40982 and 50001 in EEHV4B(Baylor). Furthermore, all of those same signals and putative alternative forms are fully conserved in EEHV4A(NAP22).

## DISCUSSION

There were three major goals in determining the complete genome DNA sequence of EEHV4. The first was to learn more about the nature of this novel class of elephant herpesviruses and the range of genes and genetic variation that the different EEHV species and subtypes display. The second was to further address the question of whether the entire *Proboscivirus* genus would be best classified as just an outlier member of the betaherpesviruses or instead as the prototype of a distinct subfamily of the mammalian herpesviruses (the *Deltaherpesvirinae*) separate from the alpha-, beta-, and gammaherpesvirus subfamilies (11, 18, 24, 35). Third, questions had arisen not only about whether the AT-rich and GC-rich branches of the EEHVs might also be sufficiently diverged to justify separate genus status but also more generically about the nature, origins, and significance of this commonly encountered tendency among some other herpesvirus groups as well of trending toward extremely high GC content.

The intact EEHV4 genome did prove to have many differences from the AT-rich branch viruses in a manner that is fully consistent with the predicted 30 to 35 million years since their last common ancestor as judged from the initial 5 kb of data (11). Some major features of these differences, especially relating to the extraordinarily high GC bias found in many coding regions and the novel enlarged repetitive Ori-Lyt domain, are described in detail here, whereas several other unique features relating especially to the enriched A-plus-T tracts in noncoding domains and the genus- or subfamily-specific sets of novel gene families and captured host genes, some of which are common to both the AT-rich and GC-rich branches, whereas others represent characteristic differences between them, are described in detail in the accompanying paper by Ling et al. (27). Finally, we also showed here that even just the first two independent strains of EEHV4 examined display a significant level of localized EEHV4A-4B chimerism, with at least two of the apparent four such domains (CD-I, CD-II, CD-III, and CD-IV) being quite similar to the patterns described previously for both EEHV1A-1B and EEHV5A-5B subtype pairs. Understanding such subtype and strain divergence patterns will be a critical factor if vaccination by nonpathogenic strains and routine monitoring of active infections are to become useful future management tools for EEHV hemorrhagic disease.

Our findings here of an extraordinarily high GC-rich bias within the wobble codon positions of most but not all EEHV4 genes and the contrasting feature of numerous AT-rich sequence tracts lying within the intergenic domains in all of the EEHVs raise the question of whether other herpesvirus genomes with similar overall GC contents might show similar features. In fact, HCMV, mouse CMV (MCMV), and the two highly diverged rat CMV [RCMV(M)] and RCMV(E)] genome types do all have a similar overall base composition of around 57 to 60% GC content. The only previous attempt that we are aware of to evaluate GC content patterns in those genomes was the work of Brocchieri et al. (36), which used a measure referred to as S content differences in the three different reading frames to attempt to identify or clarify additional potential coding genes in MCMV and RCMV(M). As shown by Geyer et al. (37), a small subset of those proposed corrected annotations were indeed later found to also be conserved in RCMV(E).

However, the major features of the S profiles shown in the paper by Brocchieri et al. show similarity to the GC wobble position biases that we described here for the EEHV4 genome. In fact, although those authors did not expressly say so, the S values do serve as a measure of wobble position GC biases, which are also fairly common in many of the core genes of those *Muromegalovirus* genomes. For example, in MCMV, a total of 40 genes, mostly located within the central core domains between M33 and M105, plus M23, M27, M115, and M139 to M143, show this feature with wobble position GC biases approaching or exceeding 90%, and up to a dozen more genes either do so over at least half of their protein coding regions or exceed 75 to 80% bias. On the other hand, more than 70 other genes, mostly mapping toward the ends of the genome, instead show much less or no evidence for wobble position bias. Notable examples of the latter include M122 (IE1), M123 (IE2), M74 (gO), and the N-terminal half of M55 (gB).

The MCMV genome is also very similar to HCMV in having at least three large AT-rich noncoding domains located around the Ori-Lyt, 5-kb stable intron, and upstream MIE loci, but with no more than five or so smaller intergenic domains that resemble the very large set in EEHV4 that have GC contents below 50% with a surfeit of AT-rich tracts. Overall, these results engender some suspicion that in high-GC herpesvirus genomes, the genes with high GC-rich wobble position bias generally tend to be well-established lytic cycle viral genes that function during late-lytic stages of virus infection, whereas those genes without any significant GC bias may tend to be those that function at immediate-early times and during latency, for example, and therefore need to more closely resemble host genes or, alternatively, may be genes that have been acquired relatively recently or evolved unusually rapidly.

A significant issue about EEHV pathogenicity that is not yet resolved is knowing whether the apparent observed much greater involvement of EEHV1A rather than EEHV1B, EEHV4, or EEHV5 in lethal disease in Asian elephant calves reflects different pathogenesis mechanisms or efficiency *per se* or perhaps simply reflects a much greater prevalence and universality of the former over the latter. It is also possible that the other EEHVs, including EEHV4, are just as ubiquitous in Asian elephant hosts as is EEHV1A but instead exhibit a tighter and less frequently reactivated shedding relationship (thus appearing to be less abundant overall). Furthermore, whereas the overall detection rates for EEHV1B and especially EEHV4 and EEHV5 in asymptomatic Asian elephants have been quite low in random trunk wash shedding studies, the fact that all four viruses have nevertheless swept through most of both the adults and juveniles present in the closely monitored Texas zoo herd over a 5-year testing period does tend to imply that infection by EEHV1B, EEHV4, and EEHV5 might also be just as ubiquitous in all Asian elephant populations as is EEHV1A. Once the differences or otherwise in pathogenesis of EEHV1, EEHV5, and EEHV4 and their respective A and B subtypes are better understood, detailed comparisons of the sequences and gene contents of all six genome types will hopefully provide important and useful information for future diagnosis, serological evaluation, and potential vaccine-based or other antiviral approaches to monitoring and controlling EEHV-associated hemorrhagic disease.

## MATERIALS AND METHODS

### Source.

The sequenced DNA came from a trunk wash sample collected from a 4-year-old Asian elephant that had experienced a mildly symptomatic episode of EEHV4 PCR-positive viremia starting 2 September 2014 and then presented with a transient high-level trunk wash shedding beginning in October 2014. Clinical details of the diagnosis, case history, and treatment of the case have been published under animal no. 2 in the work of Fuery et al (20).

### Library preparation and DNA sequencing.

Illumina paired-end libraries were prepared according to the manufacturer’s protocol (Illumina Multiplexing_SamplePrep_Guide_1005361_D) with modifications as described in the BCM-HGSC protocol (https://www.hgsc.bcm.edu/content/protocols-sequencing-library-construction). Briefly, 190 ng of DNA was sheared into fragments of approximately 200 to 300 bp with the Covaris E210 system followed by end-repair, A-tailing, and ligation of the Illumina multiplexing PE adaptors. Ligation-mediated PCR (LM-PCR) was performed for 6 to 8 cycles using the Library Amplification ReadyMix, which contains KAPA HiFi DNA polymerase, and universal primer IMUX-P1.0 and IMUX-P3.0. Purification was performed with Agencourt AMPure XP beads after enzymatic reactions. Size distribution of the LM-PCR products was determined using the Lab Chip GX electrophoresis system (PerkinElmer), and quantification was performed by gel analysis using AlphaView SA version 3.4 software.

Library templates were prepared for sequencing using Illumina TruSeq reagents and protocols. Briefly, the libraries (or library pools) were denatured with sodium hydroxide, diluted to 6 to 9 pM in hybridization buffer, and then loaded on a lane of a HiSeq flow cell. The libraries then underwent bridge amplification to form clonal clusters, followed by hybridization with the sequencing primer. Sequencing runs were performed on the Illumina HiSeq 2000 platform using the 2 × 100 run format.

### Illumina read processing and assembly.

A total of 416 million reads (42 Gb) were produced using the Illumina HiSeq sequencing technology. These raw reads were processed using the SeqPrep program (https://github.com/jstjohn/SeqPrep) to remove adaptor sequences. Low-quality sequences (with quality less than or equal to 2) at the ends of the reads were trimmed using an in-house script. The trimmed reads were mapped to the African elephant reference genome (Loxafr3.0) using BWA (38) to detect sequences with greater than 95% match, and such mapped reads were assumed to be of host origin and omitted from later assembly steps. The 212 million reads remaining after these processes were assembled using Velvet (39) with various k-mer sizes of either 29, 45, 63, or 75 and the following parameters: -exp_cov auto -cov_cutoff 20 -min_contig_lgth 400. The processed reads were also subsampled into smaller data sets of 20, 60, and 100 million reads. In the end, Velvet assemblies using all processed reads or using 100 million reads with the highest k-mer sizes each yielded a total of between two and five contigs that aligned to EEHV1A(Kimba) genomic DNA sequences in BLAST-N searches and added up to between 205 and 206 kb.

### DNA sequence analysis and comparisons.

After filling and joining across the several remaining small gaps between adjacent contigs by standard Sanger PCR amplification and cycle sequencing approaches, a single final consensus contig file of 205,896 bp was constructed. The assigned gene nomenclature and annotations were generated initially by BLASTP and BLASTX searches of the GenBank database for every potential ORF of greater than 80 aa that did not substantially overlap another already identified ORF. There was just a single exception to the overlap rule (E44A) within the final assignments. Subsequently, each ORF was confirmed directly for both orthologues and paralogues by amino acid identity comparisons with the corresponding ORFs in the EEHV1A(Kimba), EEHV1B(Emelia), and EEHV5A(Vijay) files, as well as with BLASTX searches of the intact EEHV4(Baylor) genome file itself. Clustal alignments and phylogenetic trees were generated in MEGA5 based on MUSCLE alignments or in MacVector 12 as described previously (18). Dot matrix diagrams showing global nucleotide alignments were generated as implemented at http://blast.ncbi.nlm.nih.gov/Blast.cgi. Simplot software (18, 40) was used to display nucleotide identity and divergence comparisons.

### Nucleotide sequence accession numbers.

The final completed annotated 205,896-bp genomic DNA sequence file of EEHV4(Baylor) is deposited at NCBI GenBank under accession number KT832477. Previous PCR data for this strain totaling 1,828 bp of unique sequence have accession numbers KR781023 to KR781037 (20). For comparative purposes, an amended version of the 178-kb EEHV1A(Kimba) genome with updated annotations has accession number KC618427 (24). Thirteen new DNA sequence files totaling close to 24.3 kb of extended data from Sanger cycle sequencing of amplified PCR loci from the prototype EEHV4(NAP22) genome have accession numbers KT832478 to KT832490 and KU147235.
