# Supplementary material for: Complete Genome Sequence of Elephant Endotheliotropic Herpesvirus 4, the First Example of a GC-Rich Branch Proboscivirus
Source: mSphere. 2016 Jun 15;1(3):e00081-15. doi: 10.1128/mSphere.00081-15 (PMC4911795; doi:10.1128/mSphere.00081-15)
Supplement: Table S1 [file sph003162095st1.pdf]

Table S1: Detailed Differences Between PCR Sequenced Regions of EEHV4A(NAP22) and EEHV4B(Baylor)

Gene/ORF Name/No.	HCMV ORF	HSV ORF	Ori-ent	Protein Name	Position No	Gene Size	Protein Size	Genbank Acc *	Status	EEHV4B(Baylor) Coordinates	Nucl Diff
EEHV4[NAP22]: Seventeen segments.					Total = 30,134-bp			Differences = 2,684 bp (= 8.8%)			
-					00001-00155	(155)		KT832478	-	15016- 17080	138
E4A	Nil	Nil	F		00156-00629	573	157		Novel		
E4B	Nil	Nil	F		00688-01581	894	297		Novel		
E4C	Nil	Nil	F		01784-02082	(299)	(100)		Novel		
E7	Nil	Nil	R		00596-00001	(596)	(199)	KU147235	Novel	20067- 26664	791
E7A	Nil	Nil	R		01569-00793	776	259		Novel		
E9	Nil	Nil	R		02567-01683	884	294		Novel		
E9A	Nil	Nil	F	vOGT	02911-04161	1260	419		Novel		
E9B-C	Nil	Nil	R		05161-04205	956	318		Novel		
E10A	Nil	Nil	R		06006-05125	881	293		Novel		
E11	Nil	Nil	R		06597-06296	(693)	(231)		Novel		
E12	Nil	Nil	R		00173-00001	(173)	(58)	KT832481	Novel	28145- 28877	12
E12A	Nil	Nil	R		00406-00140	267	88		Novel		
-					00407-00734	(327)			-		
E16	Nil	Nil	R		00054-00001	(54)	(14)	KT832482R	Novel	35518- 37300	51
E16D	Nil	Nil	R	vECTL1	00624-00067	528	175		Novel		
E17	Nil	Nil	F		00974-01309	335	112		Novel		
E17A	Nil	Nil	F		01426-01731	306	101		Novel		
E18	Nil	Nil	F		01694-01731	(47)	(16)		-		
-					00001-00214			KT832484	-	38220- 38996	15
E18C	Nil	Nil	F		00215-00502	288	95		Novel		
-					00503-00779	(276)			-		
E20B	Nil	Nil	R		00148-00001	(148)	(49)	KT832485	Novel	44302- 45485	16
E20A	Nil	Nil	F		00163-00582	420	140		Novel		
					00582-01168	(586)			-		
-					00001-00043	(43)		KT832486R	-	48950- 50247	24
E23B	Nil	Nil	R		00376-00044	333	110		Novel		
E24Bex2	Nil	Nil	R	vOX2-B	00650-00301	(349)	116		Novel		
E24Bex1	Nil	Nil	R	vOX2-B	01058-01007	(51)	(17)		Novel		
-					01059-01304	(145)			-		
E31A	Nil	Nil	R		00051-00001	(51)	(17)	KT832487	Novel	56874- 57869	107
E31Bex	Nil	Nil	R		00758-00300	458	152		Novel		
E32	Nil	Nil	R		00974-00828	(146)	(48)		Novel		
U39	UL55	UL29	F	gB	00001-01621	(1621)	(538)	KT832479R	Core	99801-101407	199
U38	UL54	UL30	F	POL	00001-01236	(1236	(412)	JN983935*	Core	103620-104855	7
U27	UL44	UL42	F	PPF	00001-00013	(14)	(3)	KT832488	Core	131010-135957	1094
E35A/U45.7	Nil	Nil	F	ORF-J	00126-00776	651	216		Novel		
U46	UL73	UL49A	F	gN	00788-01087	300	99		Core		
U47	UL74	Nil	R	gO	01863-01181	678	225		Core		
U48	UL75	UL22	R	gH	04059-01792	2268	755		Core		
U48.5/ORF-H	Nil	UL23	R	TK	04913-04019	(895)	(297)		α/γ/δ		
U50	UL	UL	F	PAC2	00001-00190	(190)	(59)	KT832480R	Core	138150-139828	186

U51	UL78	Nil	F	vGPCR1	00278-01498	1220	406		βδ		
-	-	-			01499-01681	182	-				
U60ex3	UL89ex2	UL15	R	TERex3	00316-00001	(316)	(106)	EU658935*	Core	155043-155358	0
U70	UL98	UL12	F	EXO	00001-00041	(41)	(13)	JN983097*	Core	166054-169365	26
U71	UL99	UL11	F	myrTeg	00001-00289	(289)	(96)		Core		
U72	UL100	UL10	R	gM	01603-00485	1118	372		Core		
U73/ORF-G	Nil	Ul09	F	OBP	02109-03314	(1205)	(401)		α/β2/δ		
U76	UL104	UL06	R	POR	00311-00001	(311)	(104)	JN983099*	Core	176440-177316	4
U77	UL105	UL05	F	HEL	00262-00877	(616)	(205)		Core		
U81	UL114	UL02	R	UDG	00545-00001	(545)	(142)	KT832489	Core	184361-184901	12
E40/U84.5	Nil	Nil	R	ORF-K	00264-00001	(264)	(88)	KT832490	Novel	194110-194728	2
					00263-00619	(356)			-		

*Genbank accession file numbers for all Sanger PCR sequenced loci of EEHV4A(NAP22). The four loci marked with * were published previously (11). The complete 205,896-bp genome of EEHV4(Baylor = NAP69) has accession number KT832477. Novel = Proposed *Deltaherpesvirus* specific genes and ORFs.
